# LncRNA SNHG15 regulates EGFR-TKI acquired resistance in lung adenocarcinoma through sponging miR-451 to upregulate MDR-1

**DOI:** 10.1038/s41419-020-2683-x

**Published:** 2020-07-13

**Authors:** Jiayuan Huang, Banzhou Pan, Guohao Xia, Jingni Zhu, Chenchen Li, Jifeng Feng

**Affiliations:** Department of Medical Oncology, Jiangsu Cancer Hospital & Jiangsu institute of Cancer Research & the Affiliated Cancer Hospital of Nanjing Medical University, Nanjing, Jiangsu 210009 China

**Keywords:** Biotechnology, Cancer, Cell biology

## Abstract

Lung adenocarcinoma (LUAD) is the main component of non-small-cell lung cancer (NSCLC) and causes a great health concern globally. The top priority of LUAD treatment is to deal with gefitinib resistance. Long non-coding RNAs are certified to modify gefitinib resistance in the course of tumor aggravation. The study focuses on addressing the function of small nucleolar RNA host gene 15 (SNHG15) on modifying gefitinib resistance in LUAD. Previously, NOTCH pathway is implicated in LUAD chemo-resistance. SNHG15 level was boosted following the depletion of NOTCH-1 in A549/GR and H1975/GR cells. Functional studies indicated that SNHG15 and multidrug resistance protein 1 (MDR-1) were overexpressed and possess tumor-promoting functions in gefitinib-resistant LUAD cells while miR-451 was downregulated and possess tumor-suppressive behaviors in gefitinib-resistant LUAD cells. Mechanically, the SNHG15 was cytoplasmically distributed in GR LUAD cells. In addition, SNHG15 released MDR-1 from the suppression of miR-451, leading to MDR-1 promotion. In addition, the elevation of SNHG15 could be attributed to ZEB1. Rescue assays highlighted that downstream molecules MDR-1 and miR-451 could reverse the effects of SNHG15 downregulation on gefitinib-resistant LUAD cells. SNHG15 could alter chemo-resistance of LUAD cells to Gefitinib via regulating miR-451/MDR-1, which could be inspiring findings for the advancement of chemo-therapies for LUAD.

## Introduction

Lung cancer is identified as one of the most malignant tumors, and causes countless deaths annually around the world^1^. Lung adenocarcinoma (LUAD) is the main class of non-small-cell lung cancer (NSCLC). In the last few decades, although depressing survival rate LUAD patients has been to some extent improved by drug therapies, such as the clinical applications of paclitaxel, docetaxel, gemcitabine, and vinorelbine, the poor prognosis remains a problem due to tumor metastasis or spread^2^. Therefore, it is a serious challenge for us to explore the mechanisms of drug resistance in LUAD, clarify the interactions among key regulatory targets, develop effective therapy methods, and find important ways to improving and reversing drug resistance in the field of oncology research.

In previous studies, we have confirmed that NOTCH-1, a major receptor in the NOTCH signaling pathway, affects the development of lung cancers^3,4^. Furthermore, the expression of NOTCH-1 is closely related to histopathological typing, clinical stage, differentiation, metastasis, and recurrence in clinical specimens of LUAD^5^. In addition, the researching significance of NOTCH pathway has been demonstrated in other cancers, such as breast cancer^6^, gastric cancer^7^, and esophageal carcinoma^8^.

Long non-coding RNAs (lncRNAs) are a cluster of non-coding RNAs (ncRNAs) with over 200 nucleotides and cannot code protein, which have drawn much attention in last 10 years^9^. Also, the lncRNAs with aberrant expression usually play a key role in regulating cancers, including LUAD. For illustration, lncRNA XIST facilitates metastasis and regulates EMT process in colorectal cancer^10^. LncRNA CRNDE/PRC2 gets involved in the radiotherapy resistance of LUAD via modulating the expression of p21^11^. Further, the carcinogenesis of lncRNA SNHG15 has been verified in cancers, for example, lncRNA SNHG15 promotes colon cancer through stabilizing the expression of transcription factor (TF) Slug^12^. And SNHG15 depletion represses cell proliferation and EMT via regulating the NF-κB signaling pathway in renal cell carcinoma^13^. Intriguingly, relation between SNHG15 and chemoresistance has been suggested. For instance, SNHG15 was contributing factor for temosolomide resistance and 5-FU resistance^14,15^. Nevertheless, the molecular regulation mechanism of SNHG15 in gefitinib resistance (GR) LUAD has not been reported yet.

MicroRNAs (miRNAs) also belong to ncRNAs, and miRNAs exert vital function in cancers by interacting with lncRNAs, such as lncRNA HULC contributing liver cancer development by sequestrating miR-186^16^, and lncRNA DGCR5 promoting LUAD progression through repressing miR-22-3p^17^. The expression of miR-451 was significantly downregulated in NSCLC tissues, and it was closely associated with lymph node metastasis and survival prognosis in previous researches^18^. Moreover, overexpression of miR-451 could inhibit the sensitization of lung cancer cells to cisplatin by post-transcriptional targeting inhibition of RAB14, and reverse the drug-resistant phenotype^19^. Besides, the effects of miR-451 on regulating docetaxel-resistant cells in LUAD have been illustrated^20,21^. Nevertheless, miR-451 and GR has never been associated in LUAD before.

Also, the molecular mechanism about that the NOTCH signaling pathway participates in the regulation of tumor resistance via modulating the expression of downstream ncRNA is not defined yet. Therefore, in this study, we aimed to explore the role of SNHG15 and its downstream mechanism in regulating EGFR-TKI chemoresistance phenotype formation in LUAD, which may provide some novel thoughts for LUAD treatment.

## Materials and methods

### Cell culture

LUAD cell lines A549 and H1975 cells (both EGFR WT), acquired commercially from Chinese Academy of Sciences (Shanghai, China), were allowed to grow in Dulbecco’s modified Eagle’s medium (DMEM) with 5% CO_2_ in air at 37 °C. 10% fetal bovine serum (FBS; HyClone, Logan, UT, USA) and double-antibiotics were utilized for cell culture. The Gefitinib-resistant A549 and H1975 cells (A549/GR and H1975/GR) were established in our clinical oncology laboratory center. Briefly, cells were subjected to continuous exposure to accumulating gefitinib (Sigma-Aldrich, St. Louis, USA) concentrations from 0.01 to 1 µM for about 6 months, and then the established resistant cells underwent culture for 5 days in the gefitinib-free mediums prior to later experiments.

### RNA extraction and qRT-PCR

Cellular total RNA was extracted from parent or gefitinib-resistant A549 and H1975 cells as per the guidelines of Trizol (Invitrogen, Carlsbad, CA, USA), and then was reverse-transcribed into cDNA as template for RNA detection. qRT-PCR was run with SYBR Premix EX Taq II (Takara Biotechnology, Tokyo, Japan) on Mx3000P qPCR system (Agilent Technologies, Santa Clara, CA, USA). The relative gene expression was calculated by 2^-ΔΔCt^ method, with GAPDH or U6 as reference gene.

### Western blot

Cells were planted to 6-well plates prior to collection for western blotting. The cellular protein samples were obtained from RIPA buffer (Invitrogen), separated by 12% SDS-PAGE gels, and then transferred onto PVDF membranes. 5% skimmed milk was applied to block membranes. Primary antibodies including anti-NOTCH-1 (ab52627), anti-E-cadherin (ab40772), anti-N-cadherin (ab98952), anti-Vimentin (ab92547), anti-ZEB1 (ab81972), anti-ZEB2 (ab138222), anti-EGFR (ab52894), anti-p-EGFR (ab40815), and anti-GAPDH (ab9484), as well as HRP-labeled IgG secondary antibodies, were purchased from Abcam (Cambridge, UK). Primary antibody anti-MDR-1 was obtained from Cell Signaling Technology (#13978, Massachusetts, USA). After washing in phosphate-buffered saline (PBS), membranes were immersed in ECL Prime Western Blotting Detection reagent (GE Healthcare, Chicago, IL, USA) and observed in the dark.

### Cell transfection

The specific shRNAs to NOTCH-1 (sh-NOTCH-1), SNHG15 (sh-SNHG15#1/2), MDR-1 (sh-MDR-1#1/2), ZEB1 (sh-ZEB1#1/2), ZEB2 (sh-ZEB2#1/2), and control shRNA (sh-NC), were all produced by RiboBio (Guangzhou, China). MiRNA mimics and NC mimics, miR-451 inhibitor and NC inhibitor, the pcDNA3.1/MDR-1, pcDNA3.1/ZEB1, pcDNA3.1/ZEB2, and control vectors, were all designed by GenePharma (Shanghai, China). Cells transfection with indicated plasmids was carried out using Lipofectamine 2000 (Invitrogen) for 48 h.

### Colony formation

Cultured cells were collected and plated to 6-well plates for 14 days of incubation in presence of gefitinib. After fixation in 96% ethanol, colonies were stained with 0.5% crystal violet for observation.

### EdU staining

Transfected cells in 96-well plates were cultivated with 50 μM of EdU solution (Ribobio) for 3 h and 4% paraformaldehyde for 15 min. Following treatment with 1 × Apollo® 488 fluorescent staining, nuclei were stained with DAPI in the dark. Proliferative cells were identified under fluorescent microscope (Leica, Wetzlar, Germany).

### Flow cytometry analyses

For cell cycle analysis, cells were seeded into 6-well plates, and then trypsinized and centrifuged. After washing in PBS, cells were treated with 70% of ice-cold ethanol for 2 h all night. The cell cycle detection kit (BD Bioscience, San Jose, CA, USA) was added for 30 min. For cell apoptosis analysis, cells in cold PBS were incubated with 100 μL of 1× binding buffer and 5 μL of Annexin V-PE and 7AAD (BD Biosciences) for 15 min in the dark. Both were analyzed by FACS cytometry (BD Biosciences).

### Cell migration assay

Cells in PBS were re-suspended in serum-free culture medium and then added into the upper chamber (8 μm pore size; Millipore, Billerica, MA, USA). 20% FBS-complete medium was added to the lower chamber. The migrated cells to the lower face of the filters were subjected to methanol for fixation, crystal violet for staining, and microscope for imaging.

### Immunofluorescence (IF)

Cells were pre-plated on the cultured slides for 24 h, rinsed in PBS and then fixed with ice-cold methanol-acetone for 10 min. Following blocking with 5% skimmed milk and detection with primary antibodies against E-cadherin and N-cadherin in PBS for 2 h at room temperature, secondary antibodies were added for 1 h. The slides were dyed with DAPI for 10 min and tested by confocal imaging system (Olympus, Tokyo, Japan).

### Subcellular fractionation

Nuclear and cytoplasmic RNAs were extracted in line with the invitrogen nuclear extraction protocol. Cell lysates in cell fractionation buffer were centrifuged to acquire cell cytoplasm. Then, cell disruption buffer was added for lying cell nuclei. The SNHG15 level was examined by qRT-PCR, with GAPDH and U6 as cytoplasmic and nuclear normalizations.

### Dual-luciferase reporter gene assay

The sequence fragment of SNHG15 containing the putative target sites for miR-873-5p, miR-24-3p, miR-153-3p, miR-451, miR-200a-3p or miR-141-3p were amplified and inserted into the pmirGLO Luciferase Vector, then co-transfected with miRNA mimics and NC mimics into cells. The pmirGLO-SNHG15-WT/MUT and pmirGLO-MDR-1-WT/MUT reporter vectors were formed with the wild-type or mutant miR-451 binding sites. For promoter assay, cells were co-transfected with pGL3-reporter-vector containing SNHG15 promoter and ZEB1 overexpression or silencing vectors. All of the luciferase gene activities were assayed by dual-Luciferase reporter assay system (Promega, Madison, WI, USA).

### RNA immunoprecipitation (RIP)

In light of the protocol of Magna RIP Kit (Millipore), RIP assay was conducted with human anti-Ago2 antibody or negative control normal mouse IgG antibody (Millipore). Cell lysates were incubated with magnetic beads conjugated with antibodies in RIP buffer. The finally precipitated RNAs were isolated for qRT-PCR.

### RNA pull-down

The sequences of miR-451 containing possible SNHG15 binding sites or mutant sites were biotinylated into Bio-miR-451-WT and Bio-miR-451-MUT. Lysates were mixed with biotin-labeled RNAs for 1 h, with Bio-NC as control. After adding magnetic beads for 30 min, qRT-PCR was performed to measure RNA levels.

### Chromatin immunoprecipitation (ChIP)

Cells in formaldehyde were sonicated for obtaining chromatin fragments of 200–500 bp. Immunoprecipitation was conducted with anti-ZEB1 and anti-IgG antibodies (Millipore). The finally retrieved chromatin DNA was subjected to qRT-PCR analysis.

### Xenograft models

The BALB/c nude mice (male, 4–6 weeks) weighing 18–20 g were bought from Shanghai SLAC Laboratory Animal Co. Ltd. (Shanghai, China). Animal experiments obeyed guidelines of the Institutional Animal Care and Use Committee (IACUC). A549/GR cells (5 × 10^6^) suspended in PBS (50 μL) with transfection of sh-NC or sh-SNHG15#1 were subcutaneously inoculated into right flanks of each mouse. Afterwards, mice randomly group in 4, and 2 groups were treated with gefitinib (50 mg/kg) or DMSO control 3 days, one time for 4 weeks. Tumor generation was monitored with the vernier caliper once a week. To calculate tumor volume, following formula was applied: Volume (mm^3^) = (Length × Width^2^)/2. Four weeks later, the mice were sacrificed and subsequent assays gained permission of the Animal Experimental Ethics Committee of Jiangsu Cancer Hospital.

### Statistical analysis

Quantitative data were exhibited as mean ± S.D. from three separated replications and analyzed by Prism 6.0 for Windows (GraphPad, San Diego, CA, USA). Significant differences between groups were compared with Student’s *t* test (two groups) or one-way ANOVA (multiple groups), with the threshold of *P* < 0.05.

## Results

### NOTCH signaling-related SNHG15 accelerates gefitinib-resistant LUAD cell malignant behaviors

**Fig. 1:**
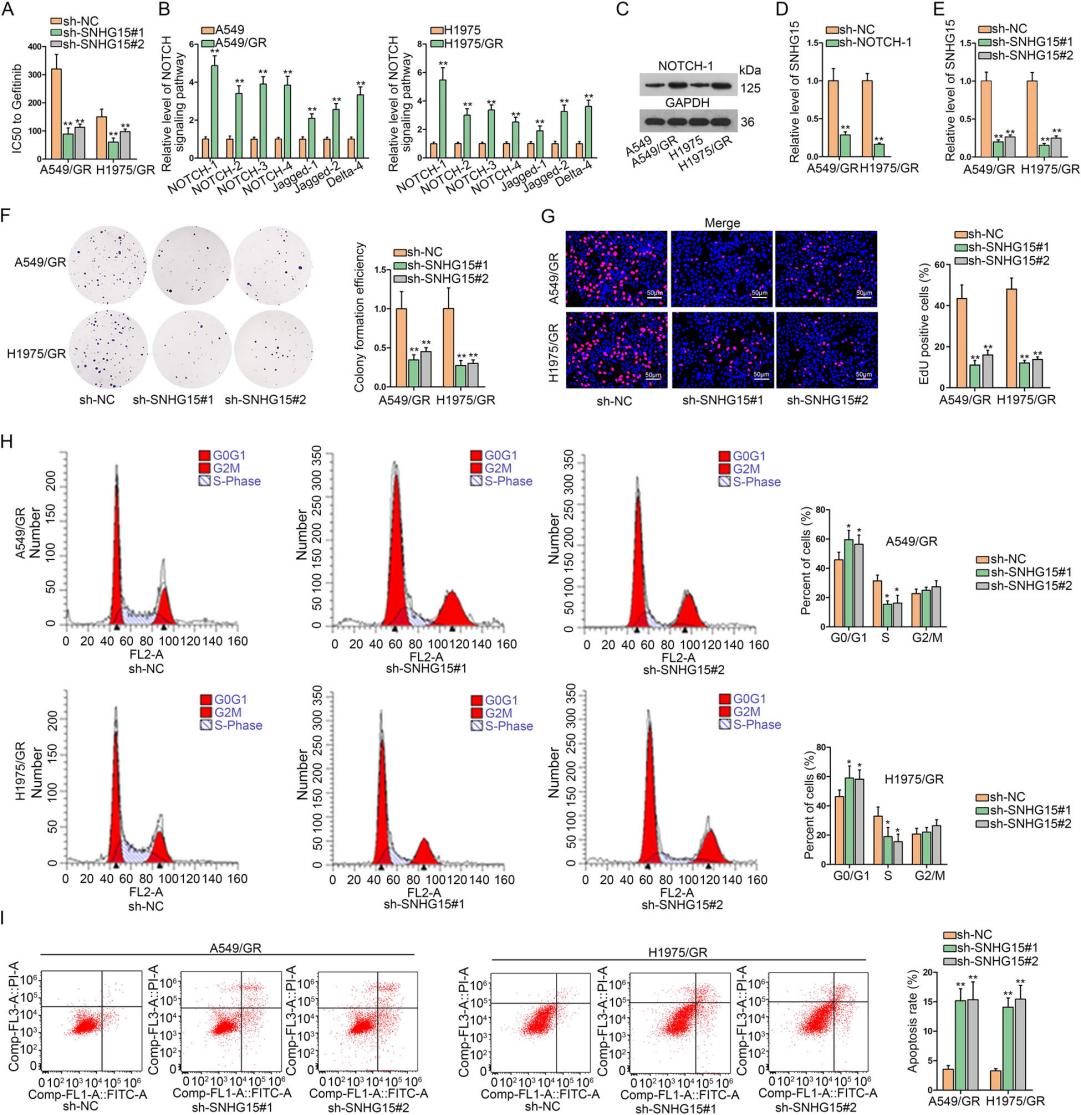
NOTCH signaling-related SNHG15 accelerates gefitinib-resistant LUAD cell malignant behaviors. **a** IC50 of A549, H1975, A549/GR, and H1975/GR was detected. **b** qRT-PCR was conducted to investigate the expression of signaling pathway receptors (NOTCH-1, NOTCH-2, NOTCH-3, and NOTCH-4) and ligands (Jagged-1, Jagged-2, and Delta-1) in human lung adenocarcinoma cells (A549 and H1975) and their relevant drug-resistant cell lines (A549/GR and H1975/GR). **c** The protein level of NOTCH-1was tested via western blot. **d** The alteration of SNHG15 expression caused by sh-NOTCH-1 was tested in A549/GR and H1975/GR via qRT-PCR. **e** qRT-PCR was used to test the interference efficiency of SNHG15. **f**–**i** The proliferation, apoptosis, and cell cycle of A549/GR and H1975/GR were investigated by colony formation assay, EdU assay and flow cytometry analysis. Data obtained from three replications were shown as mean ± S.D. *P < 0.05, **P < 0.01 indicated that differences were statistically significant.

As annotated previously, NOTCH signaling pathway is related to EGFR-TKI resistance and the clinical significance of NOTCH1 has also been highlighted previously in LUAD^3,4^. We also interrogated the relevance of NOTCH1 signaling with GR in LUAD. IC_50_ of A549/GR cells and H1975/GR cells increased versus the parental A549 and H1975 cells, confirming the acquirement of GR in both cell lines (Fig. 1a). qRT-PCR assay detected NOTCH pathway-related gene expressions. Results indicated that in comparison to parental LUAD cells, GR LUAD cells (A549/GR and H1975/GR) presented an elevated mRNA level of NOTCH pathway-related genes including NOTCH-1, NOTCH-2, NOTCH-3, NOTCH-4, Jagged-1, Jagged-2, and Delta-1, among which NOTCH1 expression was the most upregulated (Fig. 1b). Furthermore, western blot assay also confirmed that NOTCH1 was highly expressed in A549/GR and H1975/GR cells (Fig. 1c). In addition, previous studies have shown that NOTCH-1 can regulate EGFR expression in lung cancer cells^4,22^. Given that geftinib is an EGFR-TKI, we detected the influence of NOTCH-1 on EGFR expression. Consistently, we confirmed that NOTCH-1 indeed decreased EGFR level in A549/GR and H1975/GR cells (Fig. S1a). Then, we tried to detect whether SNHG15 can be affected by NOTCH-1. We observed that SNHG15 level decreased upon NOTCH-1 silence in A549/GR and H1975/GR cells (Fig. 1d), indicating that SNHG15 might participate in NOTCH-1 effect on GR in LUAD cells. Interestingly, we observed that SNHG15 knockdown failed to affect both total and phosphorylated EGFR levels in A549/GR and H1975/GR cells (Fig. S1b). Hence, we were interested whether SNHG15 could be a way for NOTCH-1 to regulate GR independent from EGFR signaling in GR LUAD cells. Hence, SNHG15 loss-of-function assay was conducted. Small hairpin RNAs against SNHG15 were constructed and transfected into A549/GR and H1975/GR cells. SNHG15 level was reduced in A549/GR and H1975/GR cells following SNHG15 knockdown (Fig. 1e). As demonstrated, we discovered that colony formation efficiency of A549/GR and H1975/GR cells was impaired following SNHG15 depletion (Fig. 1f). Similarly, EdU-positive cells were also reduced in the absence of SNHG15 (Fig. 1g). In cell cycle detection, it showed that SNHG15 depletion arrested A549/GR and H1975/GR at G0/G1 stage, while cell ratio at S-phase declined upon SNHG15 silencing (Fig. 1h). Knockdown of SNHG15 increased the proportion of apoptotic A549/GR and H1975/GR cells (Fig. 1i). Collectively, observations above highlighted that SNHG15 is crucial for GR in LUAD cells.

### Knockdown of SNHG15 retards the aggressiveness of gefitinib-resistant LUAD cells

**Fig. 2:**
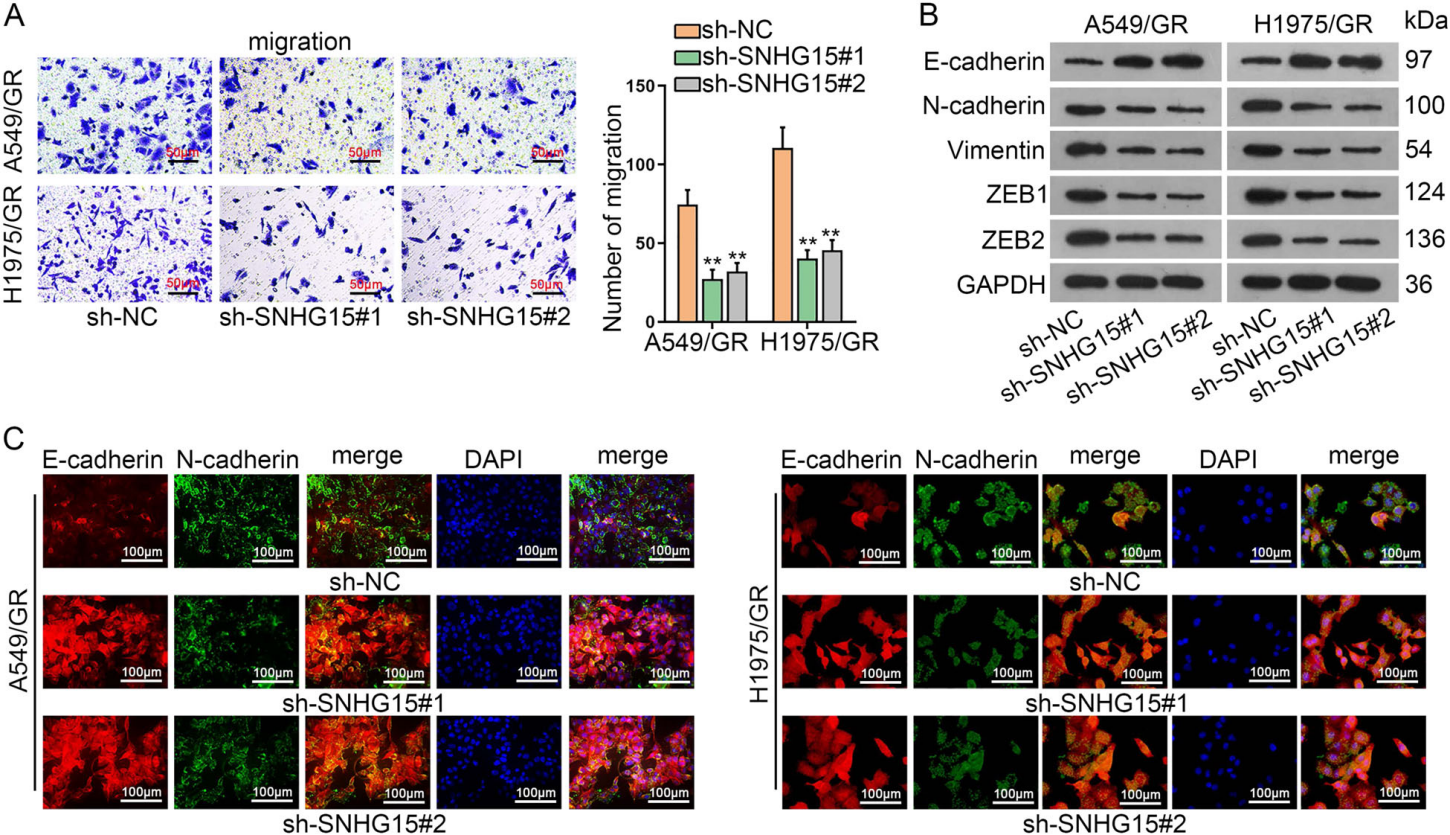
Knockdown of SNHG15 retards the aggressiveness of gefitinib-resistant LUAD cells. **a** Transwell assay was conducted to examine the migration of A549/GR and H1975/GR. **b** Western blot was implemented to test the protein levels of EMT related proteins (E-cadherin, N-cadherin, Vimentin, ZEB1, and ZEB2) in A549/GR and H1975/GR. **c** Immunofluorescence assay was conducted to investigate EMT effect of A549/GR and H1975/GR. Data obtained from three replications were shown as mean ± S.D. **P < 0.01 indicated that differences were statistically significant.

Next, we also probed the function of SNHG15 on gefitinib-resistant LUAD cell migration and EMT program. As indicated in Transwell migration assay, SNHG15 silencing prohibited migration of A549/GR and H1975/GR cells (Fig. 2a). In western blot analysis, E-cadherin was increased while Vimentin, N-cadherin, ZEB1, and ZEB2 declined after SNHG15 blockade, hinting that EMT was also hindered by SNHG15 silencing (Fig. 2b). Consistent with the data in western blot, IF also exhibited the increase of E-cadherin and decline of N-cadherin following SNHG15 downregulation (Fig. 2c). Taken together, gefitinib-resistant LUAD cell migration and EMT were limited by the downregulation of SNHG15.

### SNHG15 sponges miR-451 in gefitinib-resistant LUAD cells

**Fig. 3:**
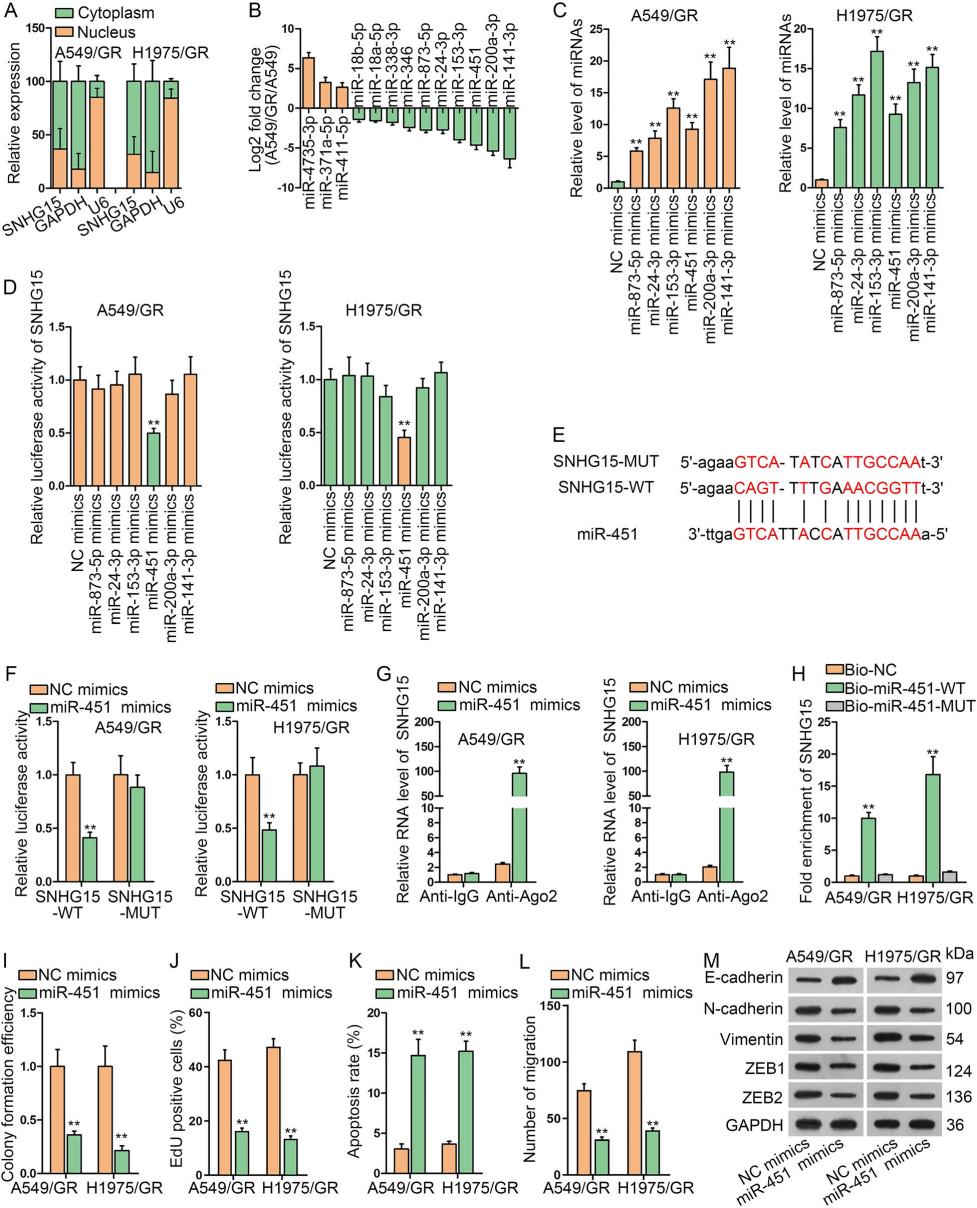
SNHG15 sponges miR-451 in gefitinib-resistant LUAD cells. **a** Subcellular fraction assay was used to locate the position of SNHG15. **b** The fold change of 13 miRNAs in A549/GR versus A549 was detected to find potential miRNA. **c** The overexpression efficiency of 6 miRNAS (miR-873-5p mimics, miR-24-3p mimics, miR-153-3p mimics, miR-451 mimics, miR-200a-3p mimics, and miR-141-3p mimics) was tested by qRT-PCR in A549/GR and H1975/GR. **d** Luciferase reporter assay was conducted to find the suitable miRNA. **e** Bioinformatics tool was used for drawing the binding site between SNHG15 and miR-451, and SNHG15-MUT sequence logo was presented. **f**–**h** Luciferase reporter assay, RIP assay and RNA pull-down assay were conducted to confirm the binding of SNHG15 with miR-451. **i**–**k** Colony formation assay, EdU assay and flow cytometry analysis were implemented to test the proliferation and apoptosis of A549/GR and H1975/GR when miR-451 was overexpressed. **l** Quantification of migrated cells in the transwell system under miR-451 overexpression. **m** The protein levels of EMT related proteins (E-cadherin, N-cadherin, Vimentin, ZEB1, and ZEB2) in A549/GR and H1975/GR were detected by western blot when miR-451 was overexpressed. Data obtained from three replications were shown as mean ± S.D. **P < 0.01 indicated that differences were statistically significant.

Afterwards, SNHG15-mediated mechanism was detected. First, the subcellular presence of SNHG15 in A549/GR and H1975/GR cells was explored. Data supported the distribution of SNHG15 majorly in A549/GR and H1975/GR cell cytoplasm, indicating the possibility that SNHG15 possessed post-transcriptional regulatory function (Fig. 3a). Starbase (http://starbase.sysu.edu.cn/starbase2/rbpMrna.php) predicted 13 miRNAs which could bind to SNHG15. Subsequently, to screen out the specific miRNA, the expressions of 13 miRNAs were compared in A549/GR and its parental cell by qRT-PCR. It turned out that 6 miRNAs (miR-873-5p, miR-24-3p, miR-153-3p, miR-451, miR-141-3p, and miR-200a-3p) were obviously downregulated (Fig. 3b). After that, these 6 miRNAs were upregulated via the transfection of miRNA mimics (Fig. 3c). Luciferase reporter assay suggested that only miR-451 mimics greatly suppressed SNHG15 luciferase activity in A549/GR and H1975/GR cells (Fig. 3d). Therefore, miR-451 was chosen. Binding sites between SNHG15 and miR-451 were acquired from online starbase (Fig. 3e). Based on this, luciferase reporter assay was performed to confirm the interaction between SNHG15 and miR-451. Results indicated that the activity of wild-type SNHG15 reporter was reduced by miR-451 mimics, while the activity of mutant SNHG15 reporter with mutations at the predicted miR-451 binding sites did not change in the presence of miR-451 mimics (Fig. 3f). RIP assay evidenced that both SNHG15 and miR-451 were enriched in the binding complex of the core protein of RNA-induced mediating complex, Ago2 (Fig. 3g). It was evident in RNA pull-down assay that Bio-miR-451-WT, but not Bio-NC or Bio-miR-451-MUT, precipitated SNHG15 (Fig. 3h). Afterwards, we monitored the function of miR-451 in A549/GR and H1975/GR cell growth, migration and EMT process. As presented in Fig. 3i, j and S2a, b, colony formation and proliferation capacities of A549/GR and H1975/GR were restrained upon miR-451 overexpression. In contrast, A549/GR and H1975/GR cell apoptosis was accelerated compared with cells in NC group (Figs. 3k and S2c). The migration of cells was weakened by miR-451 mimic versus NC mimic (Figs. 3l and S2d). As for EMT program, we unveiled via western blot that augmentation of miR-451 hampered the process of EMT (Fig. 3m). In addition, a previous study argued that miR-451 overexpression resulted in repressed EGFR signaling in breast cancer^23^, so we detected whether miR-451 affected EGFR in LUAD cells as well. Unexpectedly, miR-451 mimic failed to alter p-EGFR and EGFR levels in A549/GR and H1975/GR cells (Fig. S2e). Altogether, SNHG15 acted as a sponge of miR-451, a tumor suppressor in gefitinib-resistant LUAD cells.

### SNHG15 facilitates MDR-1 expression through serving as a ceRNA for miR-451

**Fig. 4:**
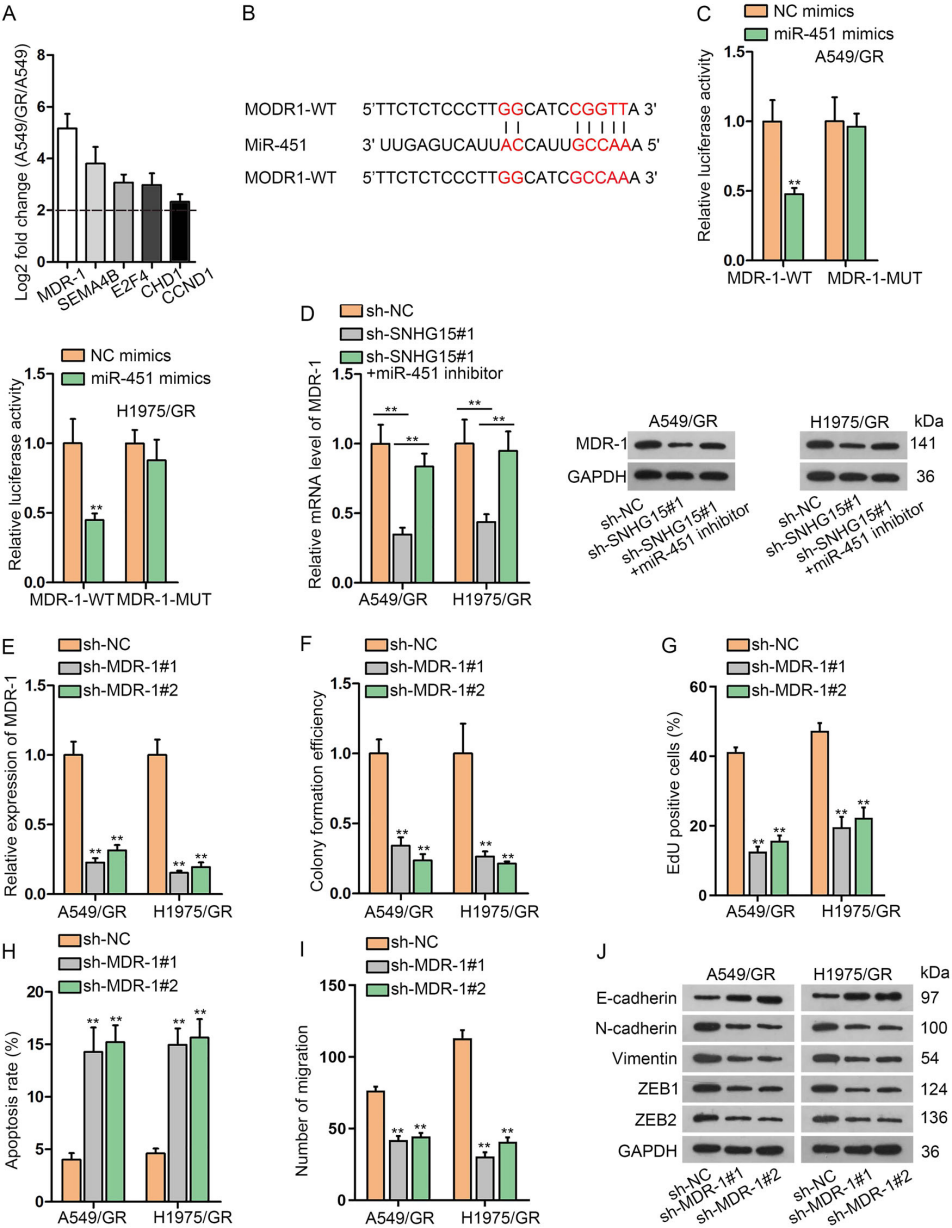
SNHG15 facilitates MDR-1 expression through serving as a ceRNA for miR-451. **a** The fold change of 5 mRNAs whose expressions were most significantly upregulated in A549/GR versus A549 was detected for searching potential mRNA. **b** The binding site between miR-451 and MDR-1 was presented according to bioinformatics tool. **c** The binding relationships of miR-451/MDR-1 and miR-451/SNHG15 were confirmed by luciferase reporter assay. **d** Western blots of MDR-1 in A549/GR and H1975/GR with suggested transfections. **e** Inhibition efficiency of MDR-1 was tested by qRT-PCR in A549/GR and H1975/GR. **f**–**i** Colony formation assay, EdU assay, flow cytometry analysis, and Transwell assay were conducted to test the proliferation, apoptosis and migration of A549/GR and H1975/GR under the influence of MDR-1 knockdown. **j** Western blot was used to test the protein levels of EMT related proteins (E-cadherin, N-cadherin, Vimentin, ZEB1, and ZEB2) in A549/GR and H1975/GR under the influence of inhibited MDR-1. Data obtained from three replications were shown as mean ± S.D. **P < 0.01 indicated that differences were statistically significant.

Bioinformatics analysis uncovered putative mRNAs for miR-451 and we profiled the expression of predicated mRNAs in H1975/GR and H1975 cells. Among top five overexpressed genes, MDR-1 (multidrug resistance protein 1) was the most overtly upregulated one in A549/GR versus A549 cells (Fig. 4a). Binding sites between MDR-1 and miR-451 were exhibited in Fig. 4b. Luciferase reporter assay described that miR-451 mimics reduced the luciferase activity of MDR-1-WT, but miR-451 mimics did not change MDR-1-MUT (mutations at the miR-451 binding sites) activity (Fig. 4c). The modulation of SNHG15 and miR-451 on MDR-1 expression was discussed and miR-451 was downregulated by transfected miR-451 inhibitor into A549/GR cells (Fig. S2f). Subsequent qRT-PCR assay pointed out that sh-SNHG15#1 suppressed MDR-1 mRNA but the introduction of miR-451 inhibitor reversed the impact. Similar trends could also be observed in western blot assay, which suggested that SNHG15-induced decline of MDR-1 protein was recovered by miR-451 inhibitor (Fig. 4d). In addition, whether MDR-1 functioned in gefitinib-resistant LUAD cells was corroborated. Firstly, MDR-1 was downregulated by shRNAs targeting MDR-1 (sh-MDR-1#1 and sh-MDR-1#2) (Fig. 4e). Colony formation ability of A549/GR and H1975/GR was attenuated by the loss of MDR-1 (Fig. 4f and S3a). Besides, the growth of A549/GR and H1975/GR was also retarded via MDR-1 deficiency (Figs. 4g and S3b). Conversely, the facilitating effect of MDR-1 downregulation on A549/GR and H1975/GR apoptosis was validated (Fig. 4h and S3c). Moreover, influenced by MDR-1 depletion, A549/GR and H1975/GR migration was suppressed (Figs. 4i and S3d). In western blot assay, we found that MDR-1 downregulation was able to attenuate EMT process in A549/GR and H1975/GR cells (Fig. 4j). However, the MDR-1 knockdown triggered no significant alteration in EGFR and p-EGFR levels in A549/GR and H1975/GR cells (Fig. S3e). These findings demonstrated that MDR-1 was protected by SNHG15 against the suppression of miR-451.

### MiR-451 or MDR-1 countervails the impacts of SNHG15 in gefitinib-resistant LUAD cells

**Fig. 5:**
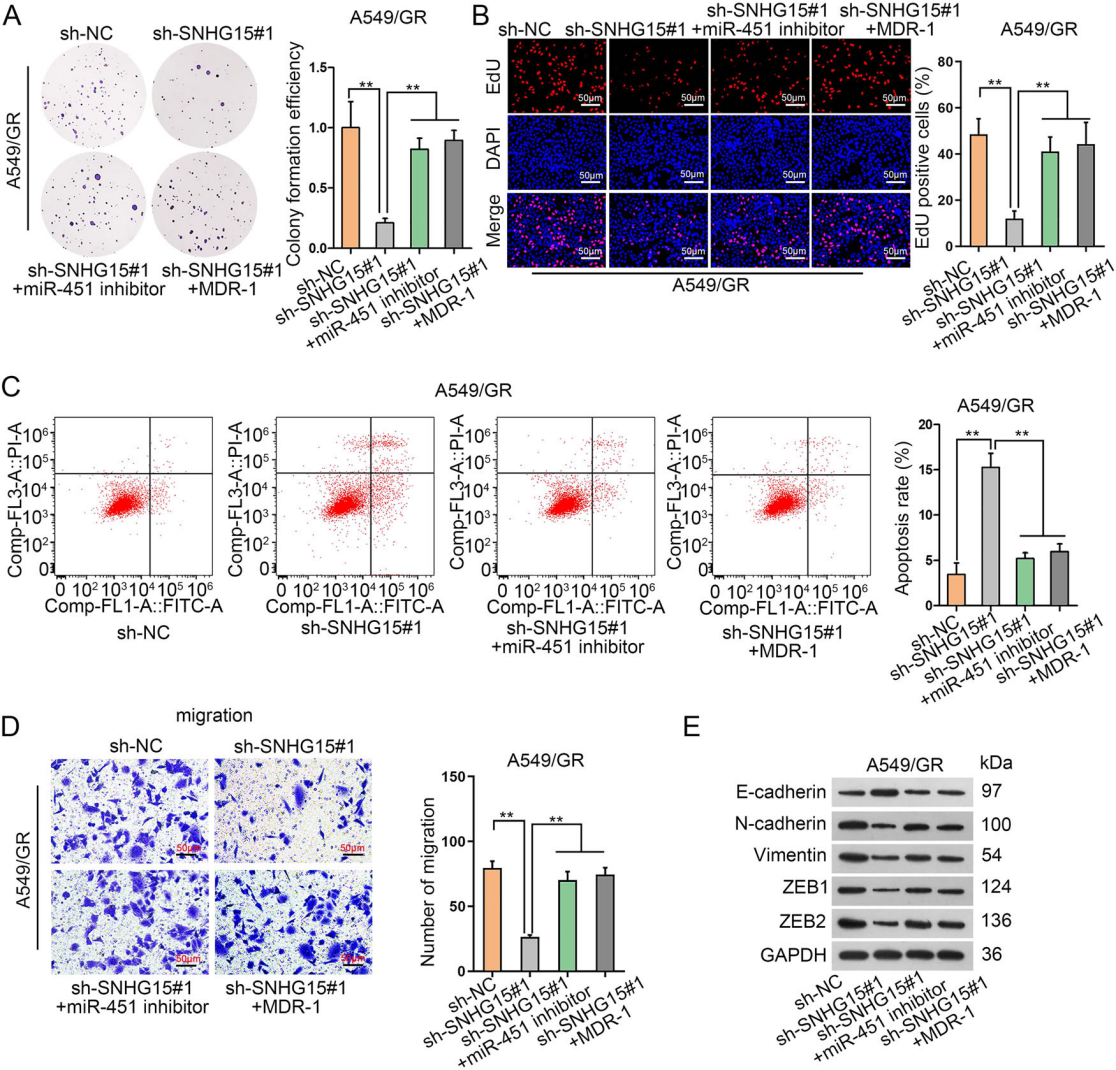
MiR-451 or MDR-1 countervails the impacts of SNHG15 in gefitinib-resistant LUAD cells. **a**–**d** Rescue experiments were implemented to test the influence of SNHG15/miR-451/MDR-1 axis on A549/GR and H1975/GR. Colony formation assay (**a**), EdU assay (**b**), flow cytometry analysis (**c**), and Transwell assay (**d**) were conducted separately to examine the proliferation, apoptosis, and migration of A549/GR and H1975/GR. **e** Rescue experiment of Western blot was used to test the protein levels of EMT related proteins (E-cadherin, N-cadherin, Vimentin, ZEB1, and ZEB2) in A549/GR and H1975/GR. Data obtained from three replications were shown as mean ± S.D. **P < 0.01 indicated that differences were statistically significant.

The ceRNA pathway of SNHG15/miR-451/MDR-1 was then functionally confirmed in cellular experiments. MDR-1 was ectopically expressed by transfecting pcDNA3.1/MDR-1 according to qRT-PCR (Fig. S3f). Consequently, the anti-proliferation property of sh-SNHG15#1 could be counterbalanced by miR-451 inhibitor or pcDNA3.1/MDR-1 (Fig. 5a, b). Flow cytometry experiments manifested that sh-SNHG15#1-caused apoptosis promotion could be alleviated by miR-451 inhibitor or pcDNA3.1/MDR-1 (Fig. 5c). A549/GR cell migration was retarded by sh-SNHG15#1, while was later facilitated via the transfection of miR-451 inhibitor or pcDNA3.1/MDR-1 (Fig. 5d). As manifested in western blot assay, the suppression on EMT caused by SNHG15 deficiency was mitigated by miR-451 downregulation or MDR-1 overexpression (Fig. 5e). In conclusion, we further confirmed that SNHG15/miR-451/MDR-1 pathway conferred gefitinib-resistant LUAD cell growth, migration, and EMT.

### ZEB1 trans-activates SNHG15 expression in gefitinib-resistant LUAD cells

**Fig. 6:**
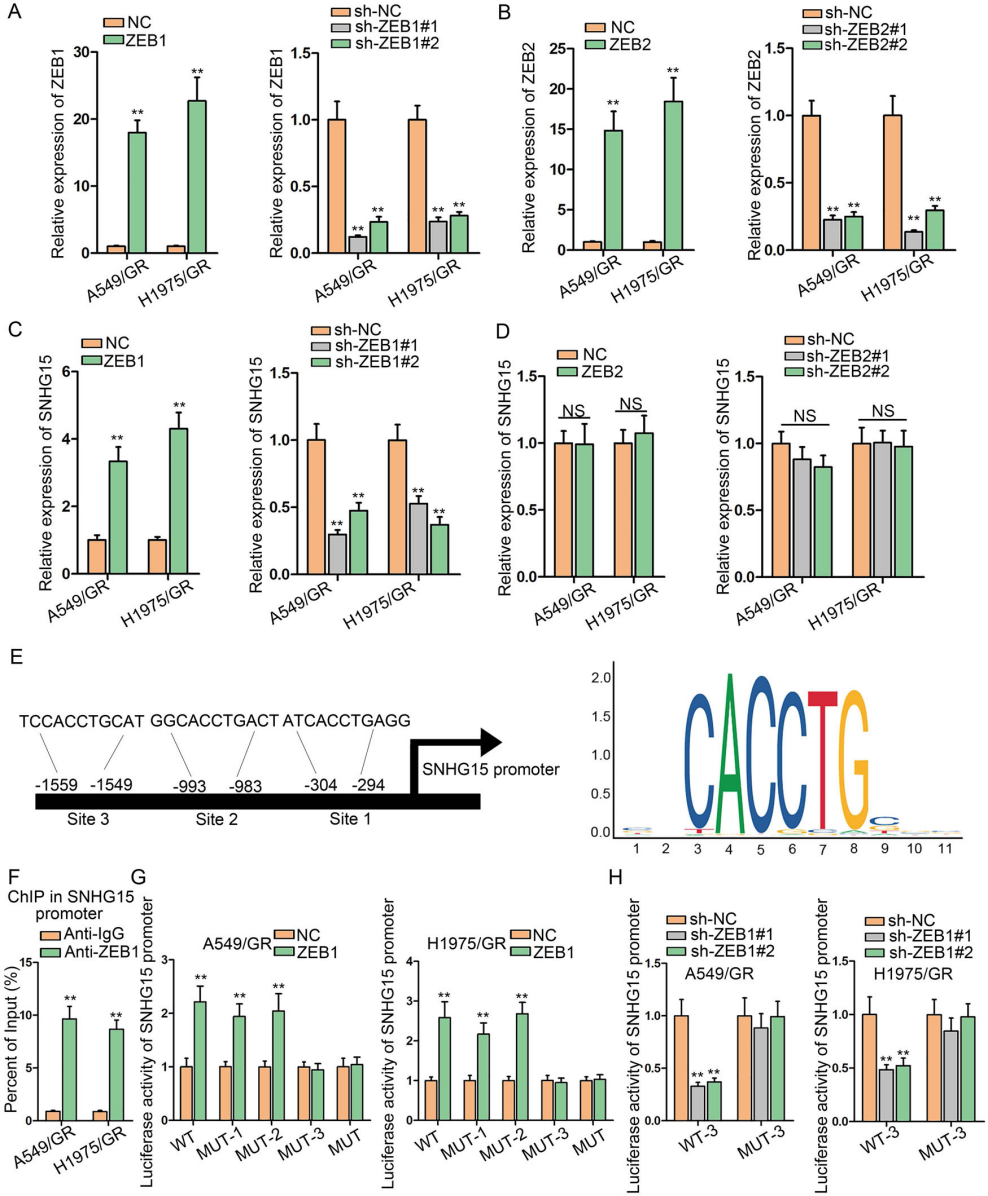
ZEB1 trans-activates SNHG15 expression in gefitinib-resistant LUAD cells. **a**, **b** qRT-PCR was used to test the expression of ZEB1/2 and inhibition efficiency of ZEB1/2 in A549/GR and H1975/GR. **c**, **d** The influence on SNHG15 expression caused by ZEB1/2 knockdown or overexpression was tested by qRT-PCR. **e** The binding sites between SNHG15 and ZEB1 and sequence logo were presented according to JASPAR (http://jaspar.genereg.net/). **f** ChIP assay was conducted to prove that ZEB1 could bind to SNHG15 promoter in A549/GR and H1975/GR. **g**, **h** Luciferase reporter assay was conducted to test the binding of ZEB1 at predicted site of SNHG15 promoter in A549/GR and H1975/GR. Data obtained from three replications were shown as mean ± S.D. **P < 0.01 indicated that differences were statistically significant. NS no significance.

We have validated that ZEB1 and ZEB2 can be positively regulated by SNHG15. Researches have supported that ZEB1 and ZEB2, recognized as TFs, can regulate transcription of target genes through binding to the promoter^24^, and several studies unveiled that ZEB1 can upregulate lncRNAs through transactivation^25,26^. Therefore, we wondered whether ZEB1 and ZEB2 could regulate SNHG15 in LUAD. To begin with, we ectopically enforced or suppressed ZEB1 and ZEB2 expressions and verified the results by qRT-PCR (Fig. 6a, b). Subsequently, the influence of ZEB1 and ZEB2 on SNHG15 expression was investigated. Overexpression of ZEB1 led to an increased level of SNHG15 and ZEB1 knockdown had opposite impacts (Fig. 6c). Apparently, either overexpression or repression of ZEB2 was unable to alter SNHG15 expression (Fig. 6d). JASPAR (http://jaspar.genereg.net/) provided the ZEB1 motif and potential sites (site 1, site 2, and site 3) in SNHG15 promoter for this interaction (Fig. 6e). Referring to ChIP experiments, in SNHG15 promoter was abundant in ZEB1 precipitates (Fig. 6f). In addition, we uncovered that luciferase activity of SNHG15 promoter was promoted in response to ZEB1-overexpressing plasmids when site 1 or site 2 in SNHG15 promoter was mutated, while the activity of SNHG15 promoter with mutations at site 3 or the mutation at all three sites did not react to ZEB1 overexpression (Fig. 6g). To substantiate that site 3 in SNHG15 promoter was responsible for the interaction with ZEB1, we carried out another luciferase reporter assay. The findings supported that sh-ZEB1#1 and sh-ZEB1#2 reduced the activity of wild-type SNHG15 promoter, while the luciferase activity could not be altered by sh-ZEB1#1 and sh-ZEB1#2 when site 3 was mutated (Fig. 6h). In summary, ZEB1 transcriptionally accelerated SNHG15 expression in gefitinib-resistant LUAD cells.

### SNHG15 contributed to GR in vivo

**Fig. 7:**
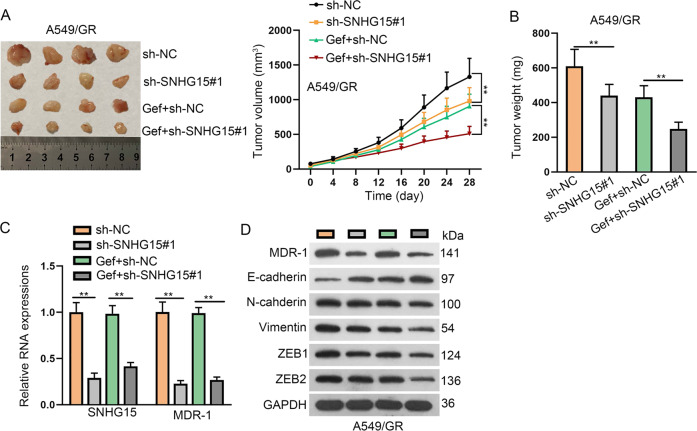
SNHG15 knockdown reversed gefitinib resistance in LUAD in vivo. **a** Mice were injected with A549/GR cells transfected with sh-NC or sh-SNHG15#1 and then mice were treated with DMSO or gefitinib. Tumor growth was measured every 4 days to draw the growth curve. **b** Tumor weight at 28 day was measured in each group. **c** qRT-PCR data of SNHG15 and MDR-1 in each group. **d** Western blot of MDR-1, E-cadherin, N-cadherin, Vimentin, ZEB1, and ZEB2 in each group. Data obtained from three replications were shown as mean ± S.D. **P < 0.01 indicated that differences were statistically significant.

Later, we unfolded animal experiments by injecting A549/GR cells into mice to generate in vivo xenografts. By monitoring tumor growth, we discovered that knockdown of SNHG15 slowed down the tumor growth of A549/GR cells in vivo (Fig. 7a). Also, gefitinib treatment repressed tumor growth, and knockdown of SNHG15 facilitated the effect of gefitinib (Fig. 7a). The tumor weight declined under SNHG15 deficiency or gefitinib treatment, and SNHG15 silence also further reduced the tumor weight that was decreased by gefitinib (Fig. 7b). Moreover, SNHG15 level and MDR-1 mRNA and protein levels declined in xenografts with SNHG15 knockdown in mice without or with gefitinib treatment (Fig. 7c, d). SNHG15 silence or gefitinib treatment caused the increase of E-cadherin and decrease of N-cadherin, Vimentin, ZEB1, and ZEB2 levels, and SNHG15 silence also further strengthened the effect of gefitinib on the levels of these proteins in xenografts (Fig. 7d). Together, SNHG15 knockdown reversed GR in vivo.

## Discussion

Previous studies established that the employment of EGFR tyrosine-kinase inhibitors (EGFR-TKIs) (such as gefitinib, erlotinib and ectinib) have achieved encouraging progress in treating NSCLC harboring EGFR mutations, unfortunately, NSCLC patients would inevitably become drug resistant^27–31^. NOTCH-1 has been reported to promote GR of NSCLC cells and epithelial-mesenchymal transition (EMT) process in gefitinib-resistant NSCLC cells^3,4^. NOTCH-1 was discovered to be upregulated in Hodgkin’s lymphoma, and its mutations were spot to serve as secondary events in some T-cell acute lymphoblastic leukemia sufferers^32,33^. Besides, NOTCH-1 has also been elucidated as critical players in several cancers, such as glioma and prostate cancer^34,35^. In lung cancer, co-expression of NOTCH-1 and vascular endothelial growth factor-A predicts poor survival^36^. In NSCLC cells, rhamnetin and cirsiliol could suppress EMT process via miR-34a-mediated inhibitive effects on NOTCH-1 level^37^. In LUAD, NOTCH-1 could activate the IGF-1R pathway to promote survival of LUAD cells under hypoxic treatment^38^. Although previous study illustrated that NOTCH-1 was conspicuously downregulated in LUAD cells and tumor tissues compared with normal cells and tissues^5^, former finding showed that NOTCH-1 exhibited elevated level in EGFR-mutant TKI-resistant NSCLC cells compared with the parental sensitive cells^39^, indicating the potential participation of NOTCH-1 in LUAD. Our study applied EGFR wild-type LUAD cells and observed that NOTCH-1 signaling pathway was activated and NOTCH-1 protein level was promoted in A549/GR and H1975/GR cells compared with the parental non-resistant cell lines, confirming the link between NOTCH-1 and TKI resistance in LUAD. In addition, several reports proved that NOTCH-1 positively regulated EGFR in NSCLC cells both in EGFR mutant (PC9 and NCI-H1650 cell lines with EGFR exon 19 deletion (DelE746-A750)) and EGFR WT cell lines (NCI-H520)^4,22^ Herein, our data consistently showed the positive regulation of NOTCH-1 on EGFR expression, indicating that NOTCH-1 might affect resistance to gefitinib in LUAD cells via EGFR signaling.

Long noncoding RNAs (lncRNAs), with a length of over 200 nt and limited protein-coding ability, have been proved to function indispensably in regulating stemness of cancer stem cells and chemoresistance^40,41^. LncRNA CASC9.5 and LINC00312 have been suggested to function as oncogenes in the development of LUAD^42,43^. Besides, lncRNA GAS5 has been discovered to facilitate gefitinib-triggered cell death in innate EGFR TKI-resistant LUAD cells via reducing IGF-1R level^44^. Existing literature pointed out that SNHG15 could exert oncogenic functions on the progression of colon cancer, NSCLC, colorectal carcinoma, and osteosarcoma via acting as a competing endogenous RNA (ceRNA) or interacting TFs^12,15,45–47^. What interested us was that SNHG15 has been linked to chemoresistance such as temosolomide resistance in glioma and 5-FU resistance in colorectal cancer^14,15^. However, SNHG15 has never been linked to gefitinib in cancer. Although SNHG15 has been related to lung cancer before^48^, the never has SNHG15 been associated with drug resistance in LUAD. Our data first verified that SNHG15 was downregulated by NOTCH-1 deficiency in gefitinib-resistant LUAD cells. Intriguingly, we found that SNHG15 cannot affect EGFR expression in gefitinib-resistant LUAD cells. This suggested that NOTCH-1 might affect GR via SNHG15 independent from EGFR signaling. Then, it was revealed that absence of SNHG15 induced an inhibition in cell proliferation, cell migration and EMT process as well as a promotion in cell apoptosis in gefitinib-resistant LUAD cells. Collectively, we first manifested that SNHG15 exhibited oncogenic properties in gefitinib-resistant LUAD cells. Also, we confirmed that SNHG15 knockdown overcome GR in vivo.

In recent years, the ceRNA regulatory network constructed by lncRNAs in cancers has drawn the attention of researchers. With the miRNA response elements, lncRNA could compete with mRNA to bind with miRNA, thereby freeing mRNA from the regulation of miRNA^49,50^. This work revealed that SNHG15 was mainly localized in the cytoplasm of A549/GR and H1975/GR cells, implying that SNHG15 might function as a ceRNA. Therefore, we searched Starbase and identified that miR-451 was closely related to NOTCH-1 by observing that miR-451 was the most upregulated under NOTCH-1 knockdown among the 13 predicted miRNAs. Our previous paper has elucidated the suppressed level of miR-451 in LUAD cells as well as the functions and molecular mechanism of dysregulated miR-451 on radioresistance of docetaxel-resistant LUAD cells^19–21^. In this study, interaction between miR-451 and SNHG15 was first uncovered by our data, and miR-451 was first linked to GR as well. Moreover, miR-451 overexpression was uncovered to restrain cell proliferation, cell migration, and EMT process as well as enhance apoptosis in resistant LUAD cells. We also provided interesting data that miR-451 failed to affect EGFR level in gefitinib-resistant LUAD cells. Former study supported the negative regulation of miR-451 on EGFR level in breast cancer^23^. This inconsistency indicated that the regulation of miR-451 on EGFR might depend on cell type and intracellular context.

Afterwards, MDR-1, which has been found to be regulated by AP-1 to influence chemoresistance of LUAD cells^51^, was unraveled as the target of miR-451. Besides, the affinity between MDR-1 and miR-451 was proved^51^. Moreover, it has been demonstrated that miR-451-MDR-1 was downstream of NOTCH-1 in regulating docetaxel-resistant LUAD cells^51^. Our data offered new evidence that NOTCH-1 and miR-451/MDR-1 axis could be linked by SNHG15 in GR. In addition, we showed that MDR-1 cannot affect EGFR expression in gefitinib-resistant LUAD cells. In consistence, an existing study showed that overexpressing MDR1 in breast cancer cells caused no significant change in EGFR level^52^. Functional assays revealed that MDR-1 posed pro-proliferation, pro-migration, pro-EMT process, and anti-apoptosis effects on gefitinib-resistant LUAD cells. In subsequence, rescue assays implied that SNHG regulates the malignancy of gefitinib-resistant LUAD cells in a miR-451 and MDR-1 dependent way. It has been well established that TFs could promote or inhibit the expression of genes, including lncRNAs^53,54^. As a key activator of EMT process, ZEB1 has been shown to activate DKK1 transcription and suppress Fbxo32 and Trim63 transcription^55,56^. Herein, we first detected the upstream mechanism of SNHG15 in LUAD. Data from our assays first suggested that SNHG15 could be transcriptionally activated by ZEB1.

This work took the first endeavor to explore the ceRNA role of SNHG15 in gefitinib-resistant LUAD cells. Taken together, NOTCH-1 confers chemoresistance of LUAD cells to gefitinib via SNHG15/miR-451/ZEB1 feedback loop, shedding a new light into the understanding of GR of LUAD cells. The weakness of this study is that the relation of miR-451 with MDR-1 has been reported by previous study, and the detailed mechanism whereby SNHG15/miR-451/ZEB1 feedback loop-regulated GR in LUAD cells independent from EGFR will be further explained in the future.

## Supplementary information

Figure S1

Figure S2

Figure S3

Supplementary figure legends
