# Supplementary figures and images for: LncRNA SNHG15 regulates EGFR-TKI acquired resistance in lung adenocarcinoma through sponging miR-451 to upregulate MDR-1
Source: Cell Death Dis. 2020 Jul 13;11(7):525. doi: 10.1038/s41419-020-2683-x (PMC7354989; doi:10.1038/s41419-020-2683-x)

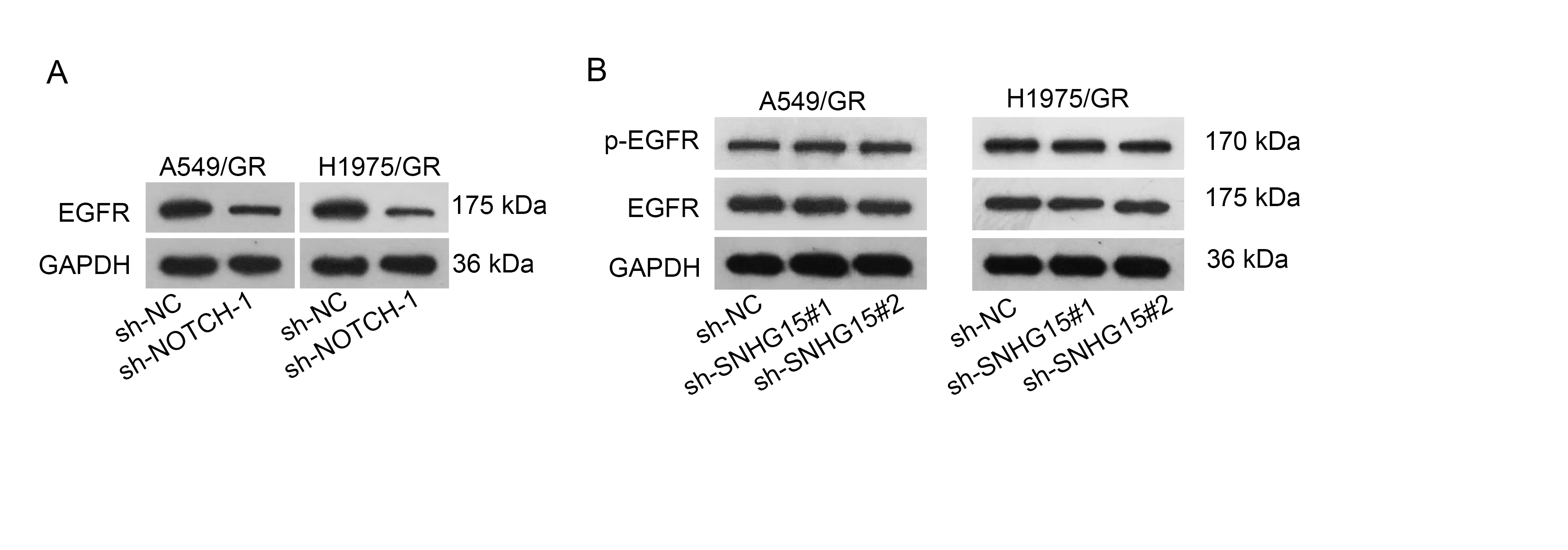

Supplement: Supplementary file 1 — Figure S1 [file 41419_2020_2683_MOESM1_ESM.tif]

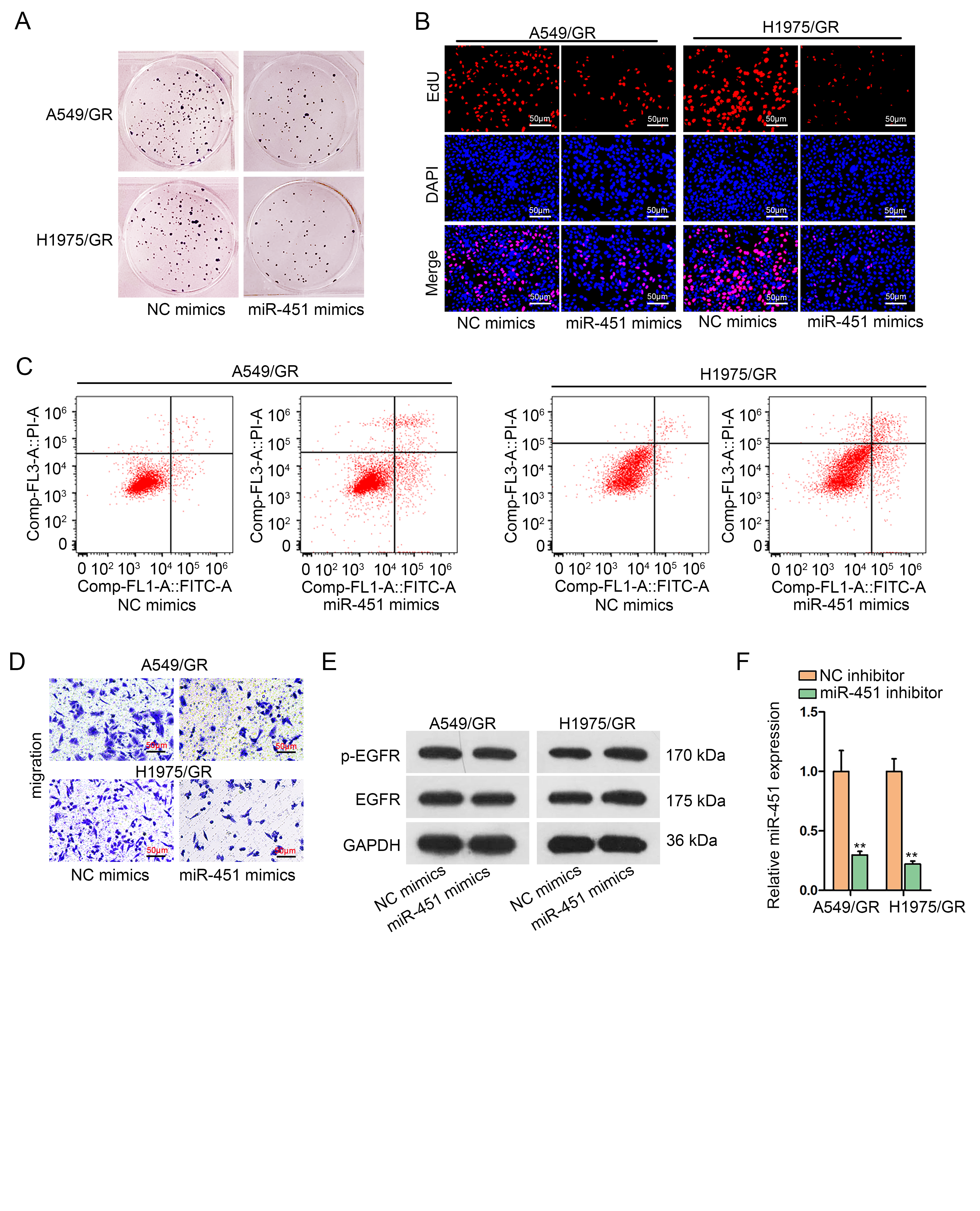

Supplement: Supplementary file 2 — Figure S2 [file 41419_2020_2683_MOESM2_ESM.tif]

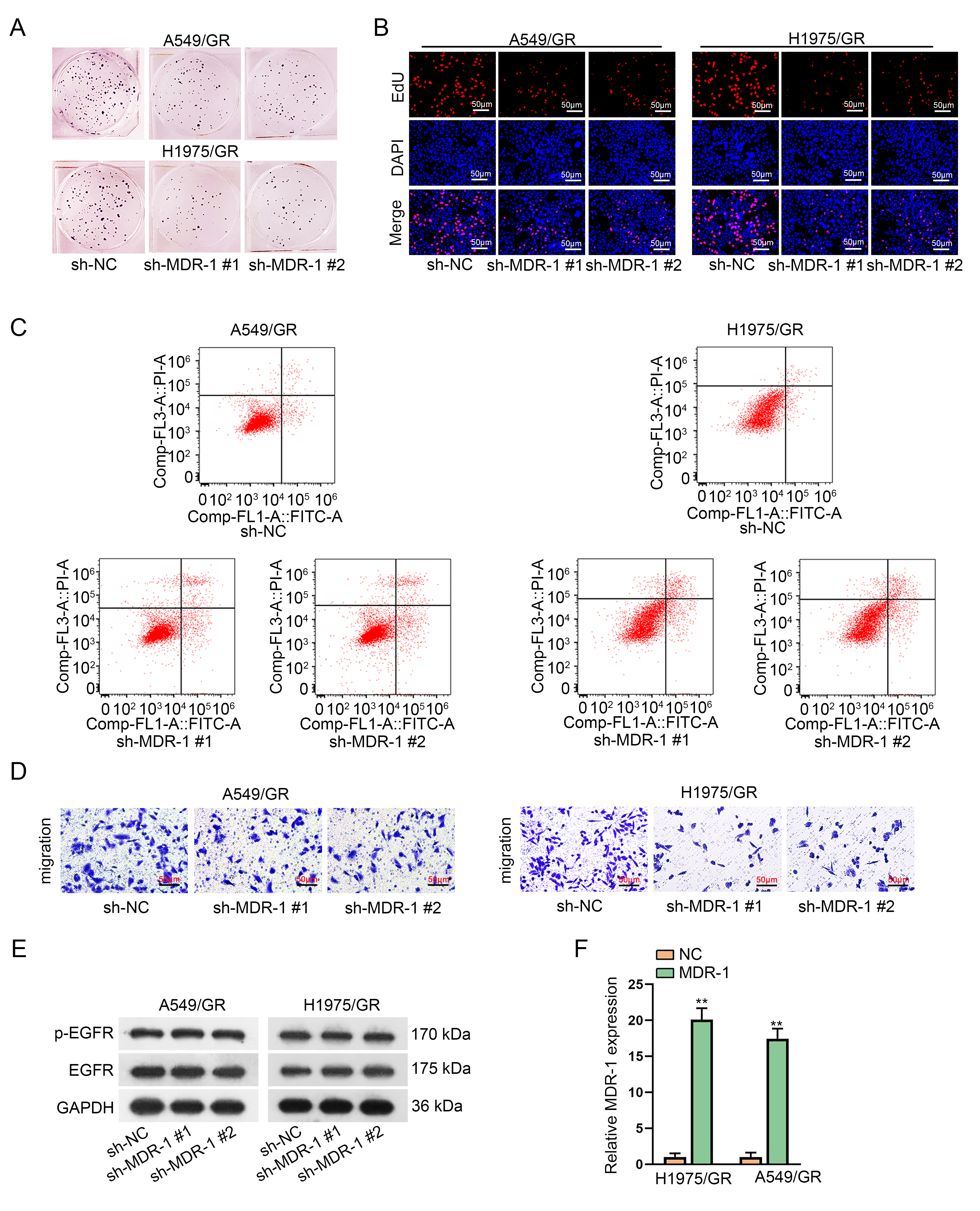

Supplement: Supplementary file 3 — Figure S3 [file 41419_2020_2683_MOESM3_ESM.tif]
