# Supplementary material for: LncRNA SNHG15 regulates EGFR-TKI acquired resistance in lung adenocarcinoma through sponging miR-451 to upregulate MDR-1
Source: Cell Death Dis. 2020 Jul 13;11(7):525. doi: 10.1038/s41419-020-2683-x (PMC7354989; doi:10.1038/s41419-020-2683-x)
Supplement: Supplementary file 4 — Supplementary figure legends [file 41419_2020_2683_MOESM4_ESM.docx]

**Figure S1 A.** Western blot of EGFR level in A549/GR and H1975/GR cells under NOTCH-1 silence. **B.** Western blot of p-EGFR and EGFR levels under SNHG15 knockdown in A549/GR and H1975/GR cells.

**Figure S2 A.** Pictures of colonies generated by A549/GR and H1975/GR cells under miR-451 overexpression. **B.** Pictures of EdU/DAPI staining in A549/GR and H1975/GR cells under miR-451 overexpression. **C.** Pictures of flow cytometry analysis of apoptotic A549/GR and H1975/GR cells under miR-451 overexpression. **D.** Pictures of migrated A549/GR and H1975/GR cells in transwell system under miR-451 overexpression. **E.** Western blot of p-EGFR and EGFR levels under miR-451 overexpression in A549/GR and H1975/GR cells. **F.** qRT-PCR results of miR-451 knockdown efficiency in A549/GR and H1975/GR cells. Data obtained from three replications were shown as mean ± S.D. **P < 0.01 indicated that differences were statistically significant.

**Figure S3 A.** Pictures of colonies generated by A549/GR and H1975/GR cells under MDR-1 knockdown. **B.** Pictures of EdU/DAPI staining in A549/GR and H1975/GR cells under MDR-1 knockdown. **C.** Pictures of flow cytometry analysis of apoptotic A549/GR and H1975/GR cells under MDR-1 knockdown. **D.** Pictures of migrated A549/GR and H1975/GR cells in transwell system under MDR-1 knockdown. **E.** Western blot of p-EGFR and EGFR levels under MDR-1 knockdown in A549/GR and H1975/GR cells. **F.** qRT-PCR results of MDR-1 overexpression efficiency in A549/GR and H1975/GR cells. Data obtained from three replications were shown as mean ± S.D. **P < 0.01 indicated that differences were statistically significant.
